# A simple-to-use clinical nomogram to predict radiation esophagitis among esophageal cancer patients receiving radiotherapy: a retrospective cohort study

**DOI:** 10.3389/fonc.2026.1900205

**Published:** 2026-08-06

**Authors:** Rui Liu, Wenyu Yang, Bingyu Li, Miaomiao Pei, Sai Wang, Xueying Gao

**Affiliations:** National Cancer Center/National Clinical Research Center for Cancer/Cancer Hospital, Chinese Academy of Medical Sciences and Peking Union Medical College, Beijing, China

**Keywords:** clinical prediction model, esophageal cancer, machine learning, nomogram, radiation esophagitis

## Abstract

**Background:**

Moderate-to-severe acute radiation esophagitis (MSARE) is a treatment-limiting toxicity of thoracic radiotherapy (RT) in esophageal cancer (EC). Early identification of high-risk patients is essential to individualize supportive care and preserve treatment continuity.

**Purpose:**

To develop and internally validate a parsimonious clinical nomogram for predicting MSARE, interpret it with SHapley Additive exPlanations (SHAP), and benchmark it against four machine learning (ML) algorithms.

**Methods:**

We retrospectively reviewed 151 EC patients who completed thoracic RT between January 2022 and December 2024. Candidate predictors were screened with LASSO regression and entered into a multivariable logistic model, visualized as a nomogram. Discrimination, calibration, clinical utility, and learning behavior were assessed by the area under the receiver operating characteristic curve (AUC), calibration plots, decision curve analysis (DCA), and a learning curve. Logistic regression was benchmarked against XGBoost, LightGBM, AdaBoost, and K-Nearest Neighbors (KNN) under stratified 5-fold cross-validation.

**Results:**

MSARE occurred in 81 of 151 patients (53.6%). Three independent predictors were retained: upper esophageal tumor (OR 7.32), hypertension (OR 2.35), and serum albumin (OR 0.85; all P < 0.05). The nomogram achieved a 5-fold cross-validated training AUC of 0.832 and validation AUC of 0.816 (95% CI: 0.676–0.949), with good calibration (*P* = 0.423) and positive net benefit on DCA. SHAP ranked albumin as the most influential predictor. Logistic regression yielded the highest validation AUC; DeLong tests showed no significant differences among models.

**Conclusions:**

An interpretable three-variable nomogram demonstrates promising internal discrimination for MSARE, yields comparable performance to four ML algorithms, and supports individualized supportive-care planning by clinicians.

## Highlights

- A parsimonious nomogram derived from three routinely measured clinical variables — upper esophageal tumor location, history of hypertension and serum albumin — enables rapid bedside estimation of MSARE risk during esophageal cancer radiotherapy, without the need for additional imaging or dosimetric data.
- With a 5-fold cross-validated AUC of 0.816, the model showed no statistically significant AUC difference compared with XGBoost, LightGBM, AdaBoost and KNN (all DeLong P > 0.05) and combines interpretability with discrimination, providing clinicians with an actionable tool for individualized supportive-care intensification.

## Introduction

Esophageal cancer (EC) accounts for an estimated 511,000 new cases and 445,000 cancer-related deaths annually, ranking 11th in incidence and 7th in mortality among all malignancies worldwide (1). Multimodality treatment incorporating definitive or perioperative radiotherapy (RT), with or without concurrent chemotherapy, has substantially improved local control and survival outcomes in both resectable and unresectable disease (2). However, owing to the anatomical proximity of the esophagus to the planned target volume, acute radiation esophagitis (ARE) remains one of the most frequent and dose-limiting toxicities of thoracic RT, with reported incidences for grade ≥ 2 disease ranging from approximately 20% to more than 50% across published EC cohorts.

According to the Radiation Therapy Oncology Group (RTOG) and European Organisation for Research and Treatment of Cancer (EORTC) toxicity criteria, RE grade ≥ 2—characterized by the requirement for continuous narcotic analgesia, marked dysphagia or worse symptoms—is defined as moderate-to-severe ARE (MSARE, 3). MSARE has been consistently linked with weight loss, malnutrition, unplanned treatment interruption, hospitalization, and, in severe cases, esophageal perforation or tracheoesophageal fistula (4–6). Treatment interruption in particular, has been associated with deteriorated local control and overall survival in radical chemoradiotherapy regimens, providing a strong rationale for early identification of at-risk patients and timely activation of supportive measures, including nutritional optimization, prophylactic analgesia, and early dietary modification.

Most existing prediction tools for radiation-induced esophagitis have been developed in cohorts of lung cancer or mixed thoracic malignancy and rely on dosimetric or imaging-derived parameters that are not available at the time of initial clinical decision-making (7–9). Comparatively few models have been derived specifically for EC, and fewer have been built solely from variables that can be obtained without additional examinations. In parallel, the recent expansion of machine learning (ML) into clinical prediction has raised the question of whether conventional logistic regression remains adequate. Systematic reviews indicate that, in low-dimensional clinical datasets, ML algorithms rarely outperform well-specified logistic models in terms of discrimination while substantially compromising interpretability (10). The recently published TRIPOD+AI guideline therefore emphasizes head-to-head benchmarking between classical and ML approaches and recommends the use of model-agnostic interpretation tools—such as SHapley Additive exPlanations (SHAP)—when prediction is intended to inform clinical decisions (11, 12).

Against this background, the present study aimed to: develop a parsimonious clinical nomogram for MSARE prediction in EC patients receiving RT based on routinely available variables; assess its discrimination, calibration, clinical utility, and learning behavior through internal validation; quantify the contribution of each predictor using SHAP; and benchmark the logistic model against four commonly used ML classifiers—XGBoost, LightGBM, AdaBoost, and KNN—using stratified 5-fold cross-validation and the DeLong test. Our goal was to deliver a transparent, externally interpretable, and clinically deployable risk-stratification tool capable of informing proactive supportive-care planning during EC radiotherapy.

## Materials and methods

### Patients selection

**Figure 1:**
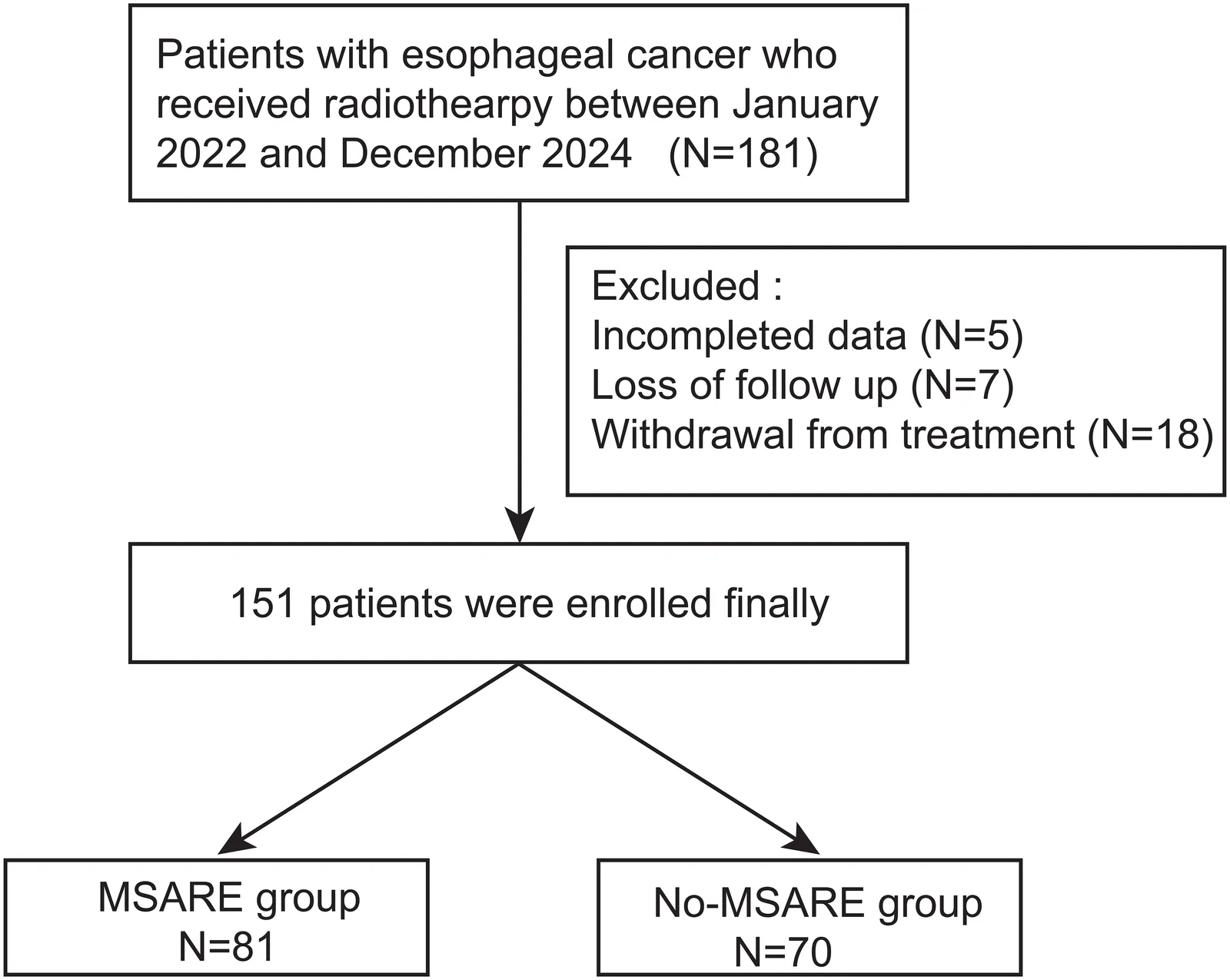
Flow chart for patient inclusion.

Patients with histologically confirmed EC who received definitive RT or concurrent chemoradiotherapy (CRT) at our institution between January 2022 and December 2024 were retrospectively reviewed. Inclusion criteria were: (i) age > 18 years; (ii) pathologically confirmed EC; (iii) completion of thoracic RT; (iv) Eastern Cooperative Oncology Group (ECOG) performance status ≤ 1; (v) expected survival > 3 months; and (vi) complete baseline clinical and follow-up information. Exclusion criteria were: (i) prior thoracic RT; (ii) history of other active malignancy; and (iii) treatment interruption due to non-toxicity reasons. After application of these criteria, 151 patients were retained for analysis (**Figure 1**).

### Sample size and number of candidate predictors

Among the 151 patients included, 81 developed MSARE. Following the conventional rule of approximately 10 outcome events per variable, this sample could reliably accommodate up to 8 candidate predictors in multivariable modeling (13, 14). LASSO regularization was used as the primary screening step, and the final model retained three predictors, yielding an events-per-variable ratio of 27. This is well within the limits recommended for robust logistic prediction model development.

### Treatment and follow-up

All patients received intensity-modulated RT (IMRT) or three-dimensional conformal RT (3D-CRT) to a total dose of 40–70 Gy delivered in 20–35 daily fractions, five days per week. Treatment plans were generated from contrast-enhanced CT simulation scans extending from the cricoid cartilage to the costophrenic angle (slice thickness ≤ 5 mm; field extension was adjusted according to the extent of tumor invasion). The gross tumor volume (GTV) encompassed the primary tumor and involved nodal disease, and the elective nodal volumes were delineated according to ESTRO-ACROP principles. The clinical target volume (CTV) was generated by adding a 5-mm isotropic margin to the GTV, and the planning target volume (PTV) was obtained by adding a further 5–8 mm setup margin to the CTV. Dose constraints for organs at risk followed RTOG recommendations. Personalized CRT, delivered either concurrently or sequentially, was administered to 99 patients; the majority received etoposide (100–120 mg/m² on days 1–3) combined with cisplatin (75 or 60 mg/m² on day 1) or carboplatin (AUC-based dosing on day 1), in cycles repeated every 21 days as recommended by current NCCN guidelines.

### Radiation esophagitis assessment

Patients were retrospectively analyzed for treatment-related toxicities at weekly intervals during RT and monthly during the first three months after RT completion. RE was diagnosed by the treating physician on the basis of symptoms, physical examination, and, when indicated, endoscopic findings, and was graded according to the RTOG/EORTC criteria. The highest observed grade during or within three months of RT was recorded for each patient. RE of grade ≥ 2 was defined as MSARE and represented the endpoint of the present study.

### Nomogram construction and internal validation

The retained predictors were used to construct a nomogram with the R rms package. Each predictor was mapped to a partial-point scale (0–100), and total points were translated into a predicted probability of MSARE. Discrimination was quantified using the AUC. Calibration was assessed visually with calibration plots — including both apparent and bias-corrected (1,000 bootstrap resamples) curves—and statistically with the Hosmer–Lemeshow goodness-of-fit test. Clinical utility was evaluated using decision curve analysis (DCA, 15). To examine whether the available sample size was sufficient for stable model fit, a learning curve was generated by progressively enlarging the training set and recomputing training and validation AUC. To render the logistic model’s predictions transparent at both the cohort and the individual-patient level, SHAP values were computed for each patient and each input variable (12, 17). SHAP assigns each variable an additive contribution to the deviation between an individual’s predicted probability and the cohort mean prediction, with the sign indicating direction (positive SHAP increases predicted risk, negative SHAP decreases it). Global feature importance was summarized as the mean absolute SHAP value across the cohort, and the distribution of feature effects was inspected with a SHAP summary (beeswarm) plot.

### Machine learning model benchmarking

To benchmark conventional logistic regression against algorithms capable of capturing non-linear interactions, four supervised ML classifiers were trained on the same three predictors: XGBoost (gradient-boosted decision trees), LightGBM (histogram-based gradient boosting), AdaBoost (adaptive boosting), and K-Nearest Neighbors (KNN, distance-based). Performance was evaluated under stratified 5-fold cross-validation, with AUC, accuracy, sensitivity, specificity, positive predictive value (PPV), negative predictive value (NPV), F1-score, and Cohen κ computed on both the training and validation folds. Pairwise differences in AUC were tested using the DeLong method (16). Clinical utility across models was further compared using DCA on the validation folds.

### Statistical analysis

Continuous variables were summarized as mean ± standard deviation (SD) or median with interquartile range (IQR) and were compared between groups using the Student t-test or the Mann–Whitney U test, as appropriate. Categorical variables were summarized as counts (proportions) and compared with the χ² or Fisher exact test. Candidate predictors of MSARE were screened with LASSO logistic regression and five-fold cross-validation; variables retained at λ_min were entered into multivariable logistic regression to compute adjusted odds ratios (OR) and 95% confidence intervals (CI). Two-sided P values < 0.05 were considered statistically significant. Analyses were performed in R 4.1.1 and Python 3.10.

## Results

### Patient characteristics and feature selection

**Figure 2:**
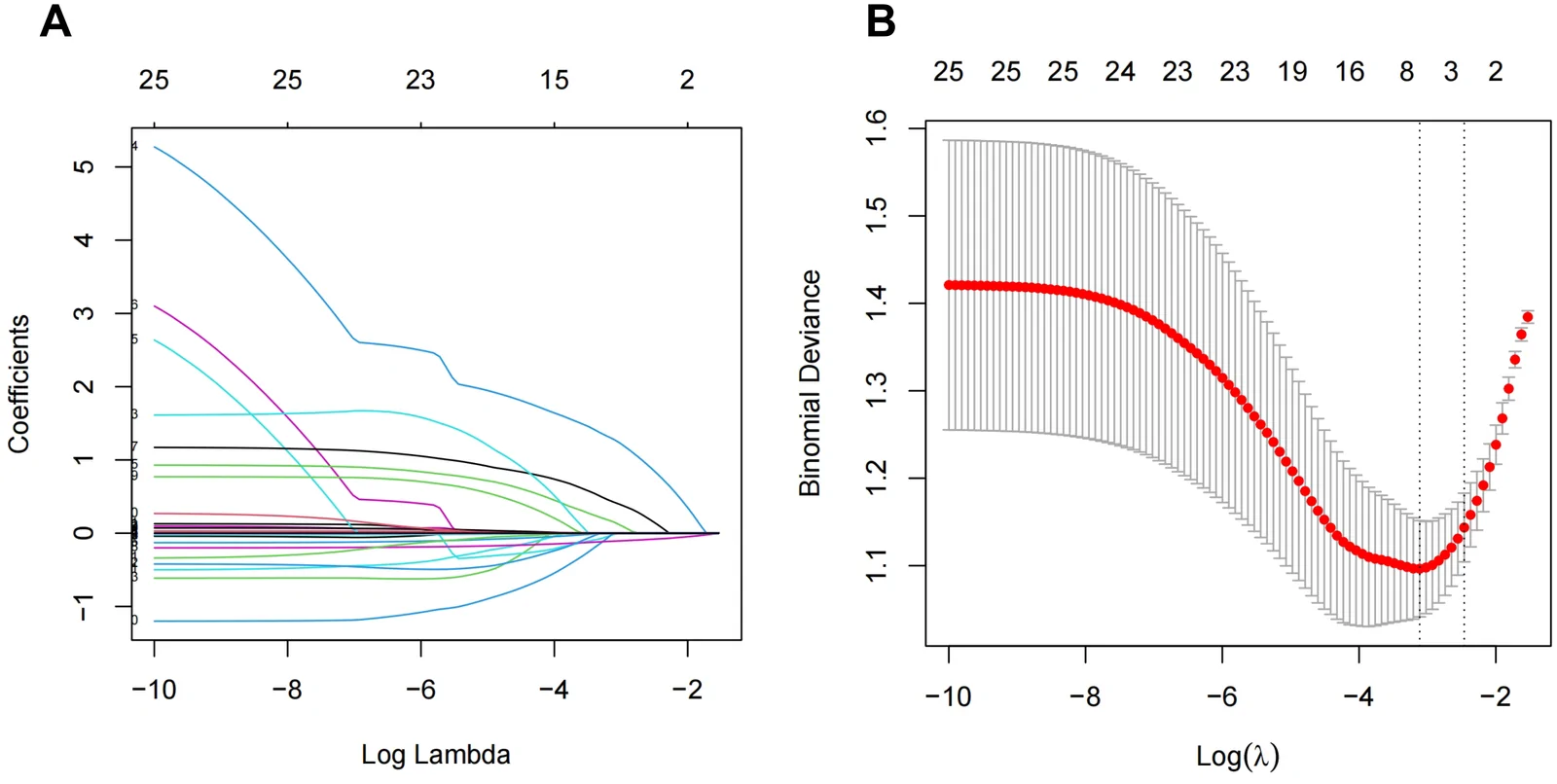
Feature selection utilizing LASSO. **(A)** The coefficient profile across different log (lambda) values. **(B)** Feature selection utilizing five-fold cross-validation with the minimum criterion. Vertical dashed lines represent the minimum and 1-SE criteria, marking the optimal lambda that yielded three nonzero coefficients.

A total of 151 patients were enrolled, comprising 121 men (80.1%) and 30 women (19.9%), with ages ranging from 18 to 89 years (median 66; IQR 59.0–72.5, **Figure 1**). MSARE developed in 81 patients (53.6%) and was absent in 70 (46.4%). Baseline demographic, clinical, and laboratory characteristics in both groups are summarized in **Table 1**. Out of 26 candidate variables, three predictors—upper esophageal tumor, hypertension, and serum albumin (ALB)—were retained on LASSO with non-zero coefficients (**Figures 2A, B**), and they were entered into the multivariable logistic regression model.

**Table 1:** Baseline demographic, clinical and laboratory characteristics of the study cohort, stratified by MSARE status.

Variable	All (n = 151)	Without MSARE (n = 70)	With MSARE (n = 81)	P value
Sex				0.565
Female	30 (19.9%)	12 (17.1%)	18 (22.2%)	
Male	121 (80.1%)	58 (82.9%)	63 (77.8%)	
Age (years)	66.0 [59.0–72.5]	64.5 [58.0–70.8]	67.0 [61.0–73.0]	0.134
Pathology				0.686
Small cell	6 (3.97%)	2 (2.86%)	4 (4.94%)	
Squamous	145 (96.0%)	68 (97.1%)	77 (95.1%)	
Upper esophageal tumor				<0.001
No	107 (70.9%)	62 (88.6%)	45 (55.6%)	
Yes	44 (29.1%)	8 (11.4%)	36 (44.4%)	
Middle esophageal tumor				0.011
No	105 (69.5%)	41 (58.6%)	64 (79.0%)	
Yes	46 (30.5%)	29 (41.4%)	17 (21.0%)	
Lower esophageal tumor				0.219
No	91 (60.3%)	38 (54.3%)	53 (65.4%)	
Yes	60 (39.7%)	32 (45.7%)	28 (34.6%)	
Hypertension				0.002
No	91 (60.3%)	52 (74.3%)	39 (48.1%)	
Yes	60 (39.7%)	18 (25.7%)	42 (51.9%)	
Diabetes				0.725
No	120 (79.5%)	57 (81.4%)	63 (77.8%)	
Yes	31 (20.5%)	13 (18.6%)	18 (22.2%)	
CHD				0.759
No	138 (91.4%)	65 (92.9%)	73 (90.1%)	
Yes	13 (8.61%)	5 (7.14%)	8 (9.88%)	
Other comorbidities				0.889
No	129 (85.4%)	59 (84.3%)	70 (86.4%)	
Yes	22 (14.6%)	11 (15.7%)	11 (13.6%)	
Smoking history				0.457
No	62 (41.1%)	26 (37.1%)	36 (44.4%)	
Yes	89 (58.9%)	44 (62.9%)	45 (55.6%)	
Drinking history				0.981
No	53 (35.1%)	24 (34.3%)	29 (35.8%)	
Yes	98 (64.9%)	46 (65.7%)	52 (64.2%)	
Clinical stage				0.515
I–II	30 (19.9%)	16 (22.9%)	14 (17.3%)	
III–IV	121 (80.1%)	54 (77.1%)	67 (82.7%)	
RT dose (cGy)	5509 (666)	5448 (673)	5561 (658)	0.298
Chemotherapy				0.131
No	52 (34.4%)	29 (41.4%)	23 (28.4%)	
Yes	99 (65.6%)	41 (58.6%)	58 (71.6%)	
BMI (kg/m²)	22.5 (3.52)	22.4 (3.36)	22.6 (3.66)	0.807
CRE (μmol/L)	63.0 [54.0–71.7]	64.4 [56.3–73.1]	61.1 [52.0–71.3]	0.226
Albumin (g/L)	35.6 [29.6–40.5]	39.0 [34.4–42.8]	31.2 [27.5–37.6]	**<0.001**
CRP (mg/dL)	0.89 [0.21–2.80]	0.69 [0.19–2.06]	1.07 [0.29–2.97]	0.266
WBC (×10^9^/L)	4.46 [3.53–5.76]	4.46 [3.58–5.85]	4.29 [3.39–5.74]	0.603
Neutrophils (×10^9^/L)	3.43 [2.59–4.96]	3.60 [2.73–5.16]	3.25 [2.38–4.70]	0.175
Lymphocytes (×10^9^/L)	0.40 [0.27–0.64]	0.38 [0.28–0.62]	0.42 [0.26–0.64]	0.792
Monocytes (×10^9^/L)	0.42 (0.20)	0.41 (0.21)	0.44 (0.19)	0.504
PLT (×10^9^/L)	168 [120–212]	168 [121–201]	168 [119–224]	0.544
HGB (g/L)	120 [108–129]	122 [110–131]	117 [107–126]	0.372

Upper esophageal tumor location, middle esophageal tumor location, hypertension and serum albumin differed significantly between patients who developed MSARE and those who did not (P < 0.05, shown in bold). Categorical variables are presented as n (%) and compared using Pearson’s χ² or Fisher exact test; continuous variables are presented as median [IQR] or mean (SD) and compared using the Wilcoxon rank-sum test or Student t-test, as appropriate. MSARE, moderate-to-severe acute radiation esophagitis; CHD, coronary heart disease; RT, radiotherapy; BMI, body mass index; CRE, serum creatinine; CRP, C-reactive protein; WBC, white blood cell count; PLT, platelet count; HGB, hemoglobin; IQR, interquartile range; SD, standard deviation.

### Development of the nomogram

**Figure 3:**
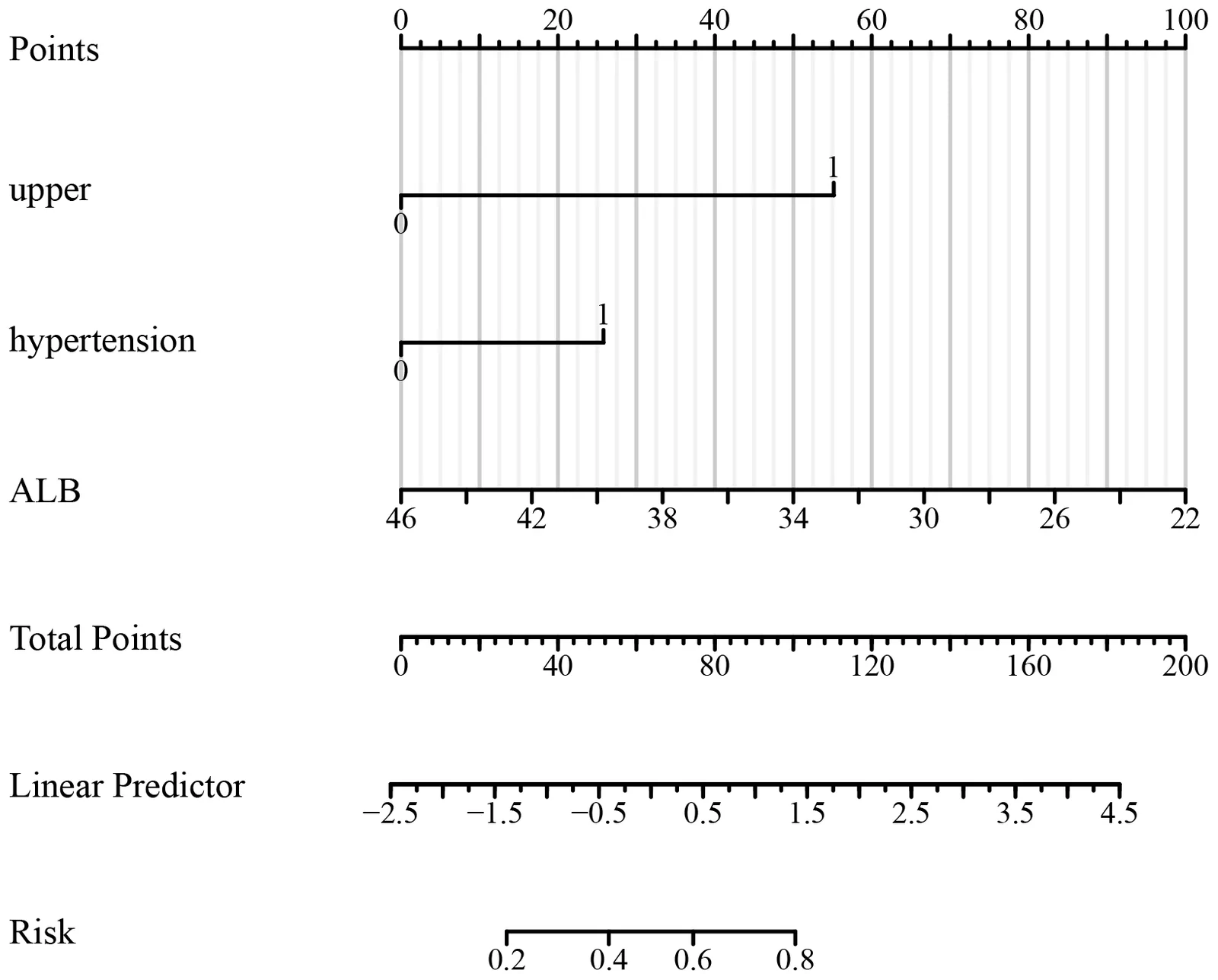
Nomogram for the prediction of MSARE. The first horizontal scale gives the number of points assigned to each value of the three predictors (upper esophageal tumor; hypertension; serum albumin). Individual scores are summed to obtain the total points, which are then projected vertically onto the bottom scale to read the predicted probability of MSARE.

Multivariable logistic regression confirmed that upper esophageal tumor (OR 7.32, 95% CI 3.00–19.90), serum albumin (OR per g/L 0.85, 95% CI 0.79–0.91), and a history of hypertension (OR 2.35, 95% CI 1.02–5.58) were each independently associated with MSARE (all P < 0.05; **Table 2**). These three predictors were integrated into a clinical nomogram, in which higher total points correspond to higher predicted probabilities of MSARE (**Figure 3**).

**Table 2:** Multivariable logistic regression analysis identifying independent predictors of MSARE.

Variable	β	OR (95% CI)	P value
Intercept	5.08	–	–
Upper esophageal tumor	1.93	7.32 (3.00–19.90)	<0.001
Hypertension	0.85	2.35 (1.02–5.58)	0.047
Serum albumin (per g/L)	-0.16	0.85 (0.79–0.91)	<0.001

Three predictors retained by LASSO regression were entered into the multivariable logistic model. The model intercept is provided to allow direct calculation of predicted MSARE probability. For serum albumin the odds ratio is reported per 1 g/L increase, indicating that each 1 g/L rise in baseline albumin reduces the odds of MSARE by approximately 15%. β, regression coefficient; OR, odds ratio; CI, confidence interval; MSARE, moderate-to-severe acute radiation esophagitis; LASSO, least absolute shrinkage and selection operator.

### Validation of the nomogram

**Figure 4:**
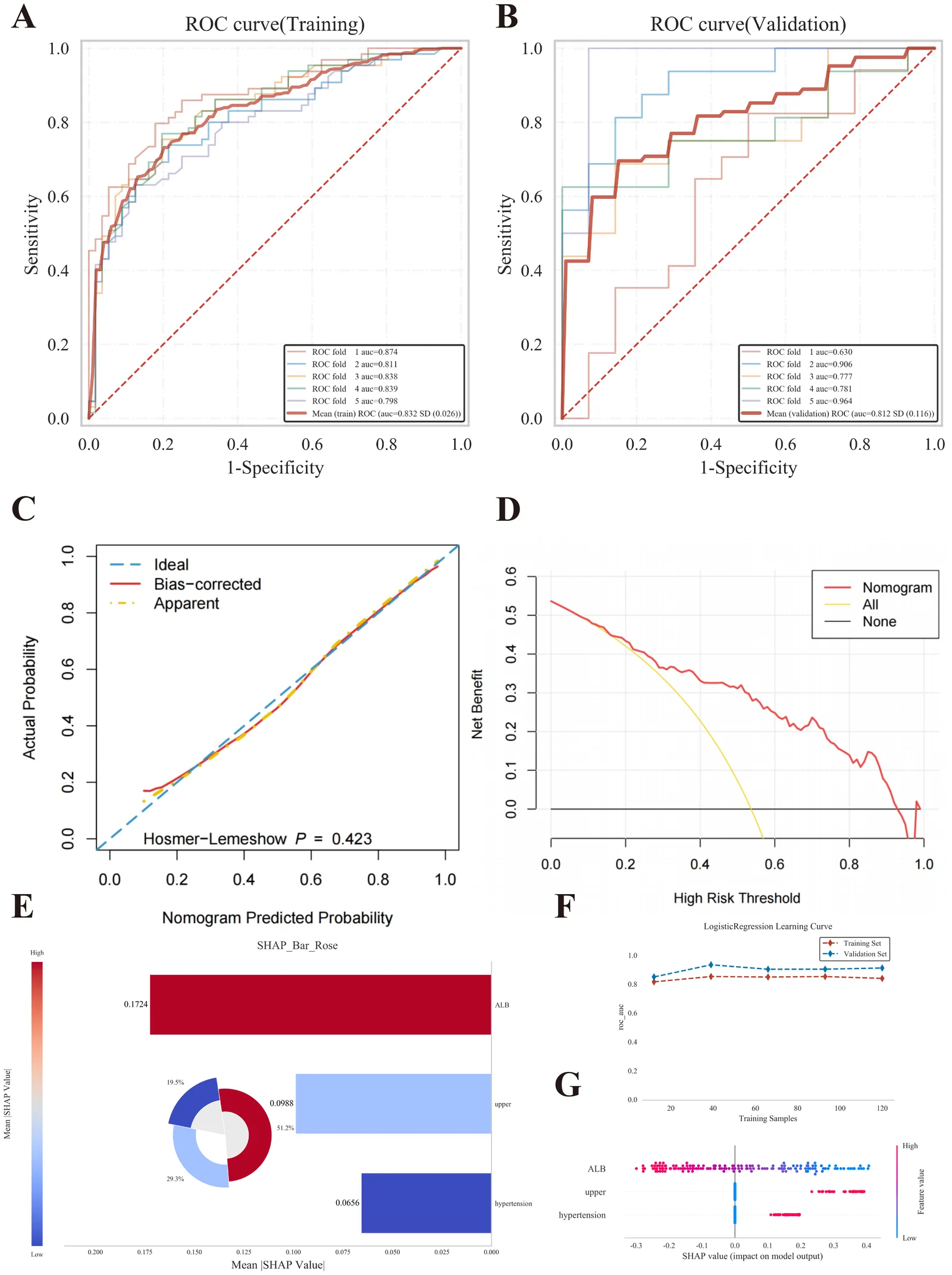
Performance and interpretation of the logistic-based nomogram. **(A)** Receiver operating characteristic (ROC) curves on the five training folds. **(B)** ROC curves on the five validation folds. **(C)** Calibration plot comparing predicted and observed MSARE probabilities. **(D)** Decision curve analysis (DCA) of the nomogram, with the “All” and “None” reference strategies for comparison. **(E)** Global feature importance computed by SHAP. **(F)** Learning curve plotting training and validation AUC against incrementally enlarged training sizes. **(G)** SHAP summary (beeswarm) plot displaying the per-patient SHAP value for each predictor.

Under stratified 5-fold cross-validation, the logistic-based nomogram showed strong and stable discrimination (**Figures 4a, b**), with a mean training AUC of 0.832 (SD 0.026; 95% CI 0.760–0.904) and a mean validation AUC of 0.816 (95% CI 0.676–0.949). The small gap between training and validation AUC (ΔAUC = 0.016) is consistent with minimal overfitting. Calibration was excellent: the bias-corrected calibration curve closely followed the diagonal of ideal calibration (**Figure 4c**), and the Hosmer–Lemeshow test confirmed good model fit (P = 0.423). At the Youden-derived optimal cut-off of 0.513, the nomogram achieved 81.5% sensitivity, 75.7% specificity, 83.5% overall accuracy, 79.5% PPV, and 77.9% NPV. Decision curve analysis demonstrated positive net clinical benefit relative to the strategies of “treat all” and “treat none” across threshold probabilities of approximately 10% to 95% (**Figure 4d**). The learning curve, which plotted training- and validation-fold AUC against incrementally enlarged training sizes, plateaued at approximately 60–80 patients with closely matched training and validation performance (**Figure 4e**), supporting the adequacy of the available sample for a stable three-variable logistic fit.

### SHAP-based feature attribution

SHAP analysis was performed on the final logistic model to quantify the global contribution of each predictor. Serum albumin emerged as the single most influential feature (mean |SHAP| = 0.172; relative contribution 51.2%), followed by upper esophageal tumor (0.099; 29.3%) and hypertension (0.066; 19.5%, **Figure 4f**). The SHAP summary (beeswarm) plot (**Figure 4g**) confirmed the expected direction of effects: lower albumin values (red) were associated with positive SHAP values and therefore higher predicted MSARE risk, whereas the presence of upper esophageal tumor and hypertension each shifted predictions toward higher risk; the directionality of all three features was concordant with the multivariable OR estimates.

### Comparison with machine learning models

**Figure 5:**
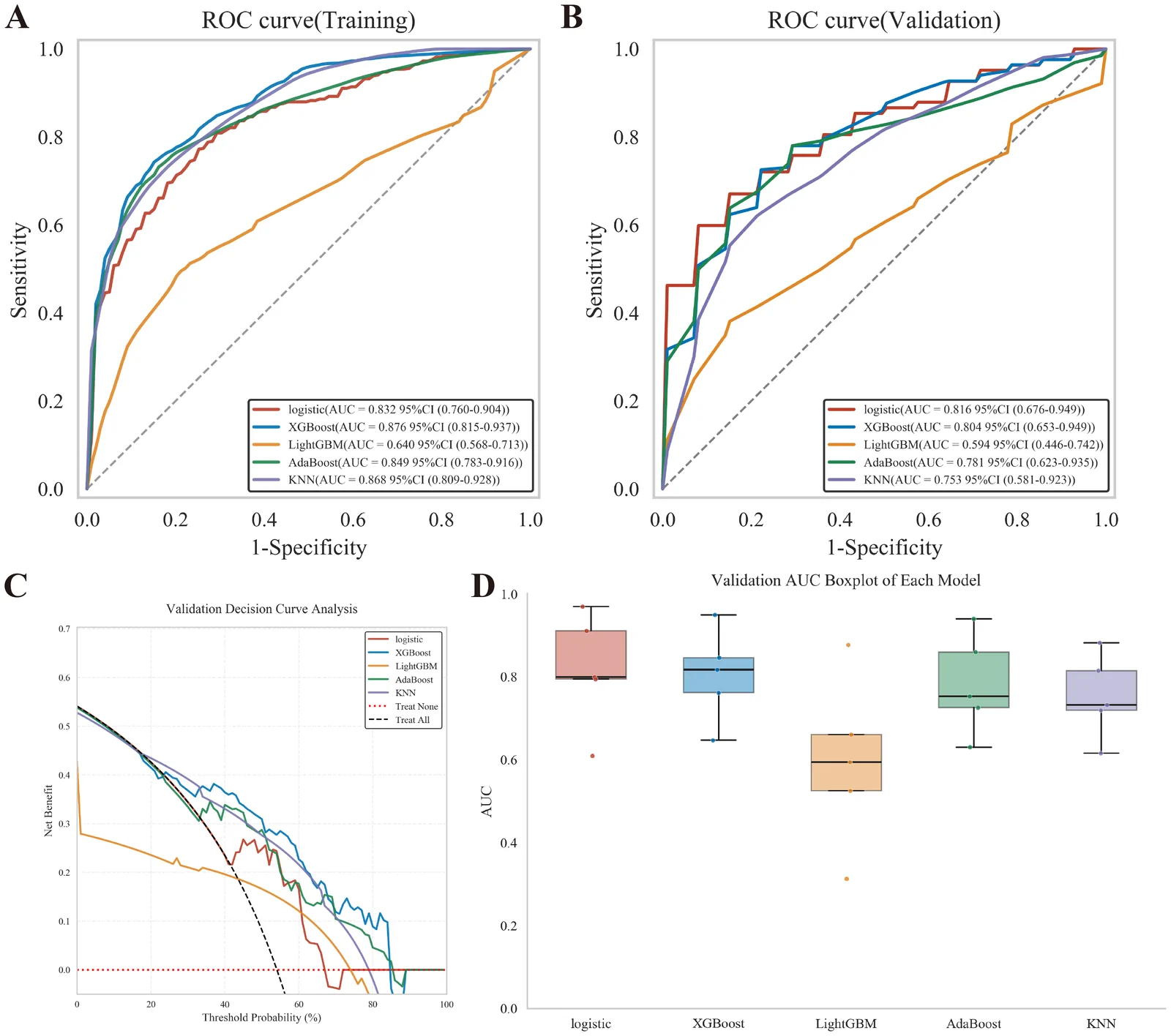
**. |** Benchmarking of logistic regression against four machine learning algorithms. **(A)** ROC curves on the training folds for logistic regression, XGBoost, LightGBM, AdaBoost, and KNN. **(B)** ROC curves on the validation folds; logistic regression yielded the highest mean validation AUC. **(C)** Decision curve analysis (DCA) of the five models on the validation folds. **(D)** Box plot summarizing the distribution of validation-fold AUCs across the five models.

On the training folds (**Figure 5a**), the five candidate models achieved AUCs of 0.876 (XGBoost), 0.868 (KNN), 0.849 (AdaBoost), 0.832 (logistic regression), and 0.640 (LightGBM). On the validation folds, the ranking shifted in favor of logistic regression: it yielded the highest mean validation AUC of 0.816 (95% CI 0.676–0.949), followed by XGBoost (0.804), AdaBoost (0.781), KNN (0.753), and LightGBM (0.594, **Figure 5b**, **Table 3**). Logistic regression also showed the smallest gap between training and validation AUC (ΔAUC = 0.016), whereas ensemble and distance-based ML models exhibited larger gaps (ΔAUC 0.07–0.12), indicating greater overfitting at the available sample size. Pairwise DeLong tests revealed no statistically significant differences in AUC between logistic regression and any of the other four models (all P > 0.05; **Table 4**). Therefore, these results should be interpreted as evidence of comparable internal discrimination. The validation AUC boxplot (**Figure 5d**) confirmed that logistic regression had the highest median validation AUC. Decision curve analysis on the validation folds (**Figure 5c**) showed marginally higher net benefit for XGBoost and KNN at threshold probabilities of approximately 30–60%, but logistic regression remained clinically useful across the relevant range.

**Table 3:** Performance of logistic regression and four machine learning models on the validation folds of 5-fold cross-validation.

Model	AUC (95% CI)	Accuracy (95% CI)	Sensitivity (95% CI)	Specificity (95% CI)	PPV (95% CI)	NPV (95% CI)	F1 (95% CI)	Kappa (95% CI)
Logistic	**0.816 (0.676–0.949)**	**0.723 (0.610–0.836)**	**0.691 (0.588–0.794)**	**0.757 (0.551–0.963)**	**0.790 (0.663–0.917)**	**0.670 (0.553–0.787)**	**0.730 (0.635–0.826)**	**0.443 (0.207–0.680)**
XGBoost	0.804 (0.653–0.949)	0.743 (0.660–0.825)	0.693 (0.588–0.797)	0.800 (0.707–0.893)	0.804 (0.721–0.887)	0.697 (0.601–0.793)	0.741 (0.659–0.823)	0.488 (0.325–0.651)
LightGBM	0.594 (0.446–0.742)	0.603 (0.478–0.728)	0.418 (0.134–0.701)	0.814 (0.609–1.000)	0.787 (0.620–0.954)	0.565 (0.443–0.687)	0.580 (0.392–0.767)	0.228 (–0.010–0.466)
AdaBoost	0.781 (0.623–0.935)	0.729 (0.655–0.804)	0.668 (0.613–0.723)	0.800 (0.697–0.903)	0.800 (0.708–0.892)	0.673 (0.605–0.741)	0.727 (0.660–0.795)	0.462 (0.312–0.611)
KNN	0.753 (0.581–0.923)	0.736 (0.677–0.794)	0.715 (0.640–0.791)	0.757 (0.627–0.887)	0.784 (0.713–0.856)	0.697 (0.639–0.756)	0.744 (0.693–0.795)	0.470 (0.347–0.592)

Logistic regression yielded the highest mean validation AUC. Values in bold represent the best-performing model for each metric. CI, confidence interval; PPV, positive predictive value; NPV, negative predictive value; F1, F1-score; KNN, K-Nearest Neighbors.

**Table 4:** Pairwise comparison of model discrimination using the DeLong test (P values).

Model	Logistic	XGBoost	LightGBM	AdaBoost	KNN
Logistic	—	0.419	0.141	0.298	0.286
XGBoost	0.419	—	0.104	0.441	0.310
LightGBM	0.141	0.104	—	0.183	0.391
AdaBoost	0.298	0.441	0.183	—	0.553
KNN	0.286	0.310	0.391	0.553	—

All pairwise comparisons involving logistic regression yielded P > 0.05, indicating that the apparent superiority of logistic regression on the validation folds was not statistically significant. KNN, K-Nearest Neighbors.

## Discussion

Definitive radiotherapy remains a cornerstone in the management of esophageal cancer, both as a curative-intent modality for unresectable disease and as part of trimodality therapy for locally advanced resectable tumors. Accurate, easily deployable prediction of MSARE is essential. In the present study, we developed a parsimonious nomogram based on three readily available clinical variables, demonstrated its robustness through internal cross-validation and learning-curve analysis, interpreted its mechanism through SHAP, and showed that it performs at least showed no statistically significant AUC difference compared with four commonly used ML algorithms on the same data.

Among the three predictors retained in the final model, upper esophageal tumor location carried the largest adjusted OR (7.32). This is anatomically and biologically plausible. The cervical and upper-thoracic esophagus lies in close proximity to the trachea, thyroid gland, and great vessels, which together reduce the geometric flexibility available for radiation-sparing beam arrangements and frequently result in incidental dose to the highly radiosensitive squamous mucosa of the laryngopharynx (18, 19). The proximal esophagus also drains into an unusually dense cervical lymphatic network; tumors of the upper third metastasize to cervical nodes in up to 60% of cases, mandating elective irradiation of the supraclavicular and lower-cervical nodal regions and consequently an elongated irradiated mucosal length (20). Experimental and clinical data further suggest that the proximal esophagus has a steeper dose–toxicity relationship than the distal segments, with proposed mechanisms including a higher density of sensory afferent fibers, a thinner muscularis mucosa, and a more limited submucosal vascular reserve in this region (21, 22). These mechanistic considerations align with our findings and reinforce the clinical strategy of contralateral esophagus-sparing planning in patients with upper esophageal tumors (18).

Hypertension was associated with a more than two-fold increased risk of MSARE in our cohort (OR 2.35). The biological basis for this association lies in the chronic vascular and inflammatory remodeling that characterizes long-standing hypertension. Endothelial dysfunction, characterized by reduced nitric oxide bioavailability, increased reactive oxygen species generation, and impaired endothelial-dependent vasodilation, predisposes vascular and perivascular tissues to amplified injury when challenged by ionizing radiation (23, 24). Hypertensive small-vessel disease limits the perfusion required to repair radiation-induced mucosal damage (25–27). Activation of the renin–angiotensin–aldosterone system in hypertensive individuals additionally promotes a pro-fibrotic and pro-inflammatory tissue microenvironment through TGF-β and NF-κB signaling, potentiating the inflammatory cascade triggered by radiation (28). In the context of concurrent chemoradiotherapy, these mechanisms may converge with chemotherapy-induced endothelial toxicity to disproportionately raise the risk of MSARE (29). Although our dataset did not permit subgroup analysis by antihypertensive class, experimental data have suggested that inhibitors of the renin–angiotensin axis may attenuate radiation-induced normal-tissue injury—an interaction worth exploring formally in future prospective cohorts.

Serum albumin emerged as both an independent predictor in the multivariable model (OR per g/L 0.85) and, on SHAP analysis, as the single most influential feature, contributing 51.2% of the model’s average attribution. Clinically, serum albumin is now recognized as more than a static index of nutritional reserve: it is a negative acute-phase reactant, downregulated in response to systemic inflammation through cytokine-mediated suppression of hepatic synthesis and capillary leak (30, 31). In radiation oncology, low pretreatment albumin reflects the dual burden of malnutrition—which is present in 60–85% of EC patients (32)—and chronic tumor-driven inflammation, both of which amplify mucosal vulnerability to radiation. Consistent with our findings, a retrospective study in EC patients showed that pretreatment albumin below 35 g/L was associated with a substantially increased risk of grade ≥ 2 RE (33). Mechanistically, albumin itself possesses antioxidant and free-radical-scavenging properties (34); under oxidative stress, it can undergo structural modification to form oxidized albumin, which loses these protective functions and contributes to pro-inflammatory signaling, microthrombosis, endothelial injury, and intensified local inflammation (35–37). Because albumin, unlike tumor location, is a potentially modifiable variable, our findings support the integration of pre-RT nutritional optimization and structured anti-inflammatory measures into the management of high-risk EC patients identified by the nomogram.

In clinical practice, for patients predicted to be at high risk, several interventions can be considered. From the radiotherapy-planning perspective, a high predicted risk may prompt review of plan quality and consideration of esophagus-sparing optimization when target coverage can be maintained. Additionally, practical interventions include pre-RT nutritional assessment and optimization, early dietary and dysphagia counseling, timely analgesic and mucosal-protective therapy, hydration or enteral nutritional support when needed, and closer symptom monitoring during treatment.

SHAP analysis adds an important interpretive layer to the nomogram. While the nomogram provides a population-level scoring system, SHAP values quantify how each variable shifts the predicted probability of an individual patient relative to the cohort average, rendering the model’s reasoning transparent at the level of the individual case. The dominance of serum albumin in SHAP attribution indicates that the strongest day-to-day variation in MSARE risk in our cohort is driven not by the binary anatomical factor of tumor location but by a continuous, modifiable nutritional–inflammatory biomarker. This observation is clinically actionable. The finding that conventional logistic regression showed no statistically significant AUC difference compared with the discrimination of XGBoost, LightGBM, AdaBoost, and KNN in our cohort is consistent with an accumulating body of evidence on the performance of ML in low-dimensional clinical datasets. In a systematic review of 71 studies comparing ML and logistic regression for clinical prediction, Christodoulou and colleagues reported no consistent advantage of ML when the sample size was modest and the number of candidate variables was limited (10). Our analysis supports this view, but it does not establish statistical superiority or formal non-inferiority of logistic regression. Together with its inherent interpretability and the convenience of being translatable into a nomogram, these considerations justified our decision to retain logistic regression as the primary model and to relegate ML results to a benchmarking role.

Several limitations should be acknowledged. First, this is a single-center retrospective analysis, and despite the application of strict inclusion criteria and validated grading scales, residual selection and information biases cannot be excluded. Second, dosimetric parameters and planning-CT-based radiomic features were not included; these variables may further refine prediction and should be explored in future iterations of the model. Third, internal validation by bootstrap and cross-validation, while informative, does not replace external validation in independent cohorts, which we are now actively pursuing through prospective multi-center collaboration.

## Conclusions

We developed and internally validated a parsimonious, interpretable nomogram for the prediction of MSARE in patients with esophageal cancer receiving radiotherapy. The model relies on three routinely available clinical variables—upper esophageal tumor location, hypertension, and serum albumin, which demonstrate promising internal discrimination, good calibration, and clear net clinical benefit on decision curve analysis. External validation is required before broad clinical implementation.

## Data Availability

The raw data supporting the conclusions of this article will be made available by the authors, without undue reservation.
